# Bacteriology and Antimicrobial Susceptibility Patterns of Childhood Acute Bacterial Conjunctivitis in Western Greece

**Published:** 2019-10-01

**Authors:** Maria-Zoi Oikonomakou, Olga E. Makri, Eleni Panoutsou, Konstantinos Kagkelaris, Panagiotis Plotas, Dionysia Garatziotou, Constantinos D. Georgakopoulos, Maria I. Eliopoulou

**Affiliations:** 1 Department of Pediatrics, ''Karamandaneio'' General Pediatric Hospital, Patras, Greece; 2 Department of Ophthalmology, Medical School, University of Patras, Patras, Greece; 3 Department of Microbiology, ''Karamandaneio'' General Pediatric Hospital, Patras, Greece

**Keywords:** Newborn, Staphylococcus, Haemophilus, Streptococcus, Anti-Bacterial Agents, Child, Infant

## Abstract

Acute bacterial conjunctivitis is a common, highly contagious infection in children and is usually treated empirically with broad spectrum topical antibiotics. In the current study we investigated bacteriology and antibiotic susceptibility patterns in childhood acute bacterial conjunctivitis in Western Greece. We conducted a retrospective analysis of presumed acute bacterial conjunctivitis cases in ''Karamandaneio'' Pediatric General Hospital of Patras, Western Greece, between February 1, 2013 and January 31, 2018. Specimens from the lower conjunctiva fornix were isolated from 191 cases and outcomes were analyzed to identify the pathogenic bacteria of acute bacterial conjunctivitis and their corresponding antibiotic susceptibility patterns. Patients were divided into 3 groups; Group A included neonates under 28 days of life, Group B children from 1 month to 2 years and Group C from 2 years to 14 years. Results revealed that *Staphylococcus* spp., *Haemophilus* spp. and *Streptococcus* spp. were the most prevalent pathogens. No significant differences in isolated pathogens were found between the age groups. Antibiotic resistance rates were higher against ampicillin, ceftriaxone, ceftazidime and sulfamethoxazole. Resistance rates to Ciprofloxacin were low while none of the evaluated isolates were resistant to vancomycin. We concluded that predominant pathogens of childhood acute bacterial conjunctivitis in Western Greece were *Staphylococcus* spp., *Haemophilus* spp. and *Streptococcus* spp. Continuous surveillance, focused in distinct geographic areas, is encouraged to prepare more precise protocols of empirical treatment.

## INTRODUCTION

Conjunctivitis is the most frequent eye disease in children, accounting for an estimated of 1-4% of visits [1]. Acute bacterial conjunctivitis constitutes its larger subset, corresponding approximately to 54-73% of all referred cases [2]. While bacterial conjunctivitis is characterized as a self-limiting condition, treatment with topical antibiotics confers several benefits. Namely, the treatment can lessen the disease duration, which can take up to 3 weeks untreated, palliate symptoms, alleviate patient discomfort, facilitate earlier resumption to normal activities, reduce the overall cost of parents’ lost productivity and children’s absence from school or day-care. However, bacterial conjunctivitis is highly contagious and rapidly transmitted, hence the treatment diminishes the risk of widespread contamination and sight-threatening complications [3]. The treatment is mainly empirical with broad-spectrum topical antibiotics [1]. Mataftsi et al. studied the current preferred practice among pediatricians, ophthalmologists and general practitioners in Northern Greece [4]. They reported that 67% of pediatricians, 73% of ophthalmologists and 25% of general practitioners prescribe an antibiotic drop empirically according to clinical examination findings without any laboratory results. 

Antibiotic resistance is the prominent factor leading to failure of the initial empirical treatment. Antibiotic resistance rates per pathogen may vary significantly in the course of time and in distinct geographic areas, mainly due to intense antibiotic use that promotes the resistant bacteria [5]. Carreras reported that in the same geographical area resistance against chloramphenicol and tetracyclines had diminished statistically significantly between 1982 and 2008, resistance against trimethoprim-sulfamethoxazole remained the same, while resistance against gentamicin, neomycin, erythromycin and tobramycin had statistically significantly increased [6]. The aim of the current study was to identify the pathogens inflicting acute bacterial conjunctivitis in children younger than 14 years in Western Greece and to assess their antibiotic susceptibility patterns. 

## METHODS

This was a single-center retrospective chart review of cases with presumed acute bacterial conjunctivitis between February 1, 2013 and January 31, 2018 in ''Karamandaneio'' General Pediatric Hospital of Patras, Western Greece. The study met the ethical guidelines of the Declaration of Helsinki and adhered to the legal requirements of the ''Karamandaneio'' General Pediatric Hospital of Patras regarding institutional review board approval for in vitro investigations of antibiotic susceptibilities of human isolates. Also a written informed consent was obtained from the participants’ legal guardians. Inclusion criteria were patients younger than 14 years with a clinical diagnosis of acute bacterial conjunctivitis in at least one eye where samples for microbiological analysis were taken. The clinical diagnosis of acute bacterial conjunctivitis was based on the presence of purulent conjunctival discharge, crusty or sticky eyelids, bulbar conjunctival injection and/or ocular surface redness. Exclusion criteria were a history suggesting allergy, ocular trauma or foreign body, Kawasaki disease, Stevens-Johnson syndrome, administration of systemic or topical antibiotics during the previous week including tear substitutes, immunocompromisation, ocular surgery during the last 6 weeks, suspicion of iritis, active ulcerative keratitis, a history of recurrent corneal erosion syndrome and signs of preseptal cellulitis. Also, not adherence to the national vaccination program constituted an exclusion criterion. Patients were divided into 3 groups; Group A included neonates under 28 days of life, Group B children from 1 month to 2 years and Group C from 2 years to 14 years. Samples for microbiological analysis were collected in a standardized fashion. More precisely, the specimens were collected under local anesthesia with Proparacaine HCl 0.5% eye drops (Alcaine, Alcon Laboratories, Inc. Fort Worth, TX) by swabbing a cotton microswab (Becton Dickinson, BBL^TM^ Culture Swab ^TM^ liquid Stuart single swab, Collection and Transport system) over the conjunctival lower cul-de-sac of the infected eye, avoiding any contact with the eyelids. All the microswabs were in a sealed sterile condition and were opened immediately before sampling. In case of bilateral infection, the eye with the most severe symptoms was selected for specimen collection. In eyes equally affected, the eye that was affected first was considered for the study. Thus, one specimen was collected from each patient. 

After sample collection, the microswabs were placed into the transport media and sent to the laboratory where they were streaked over chocolate, 5% sheep blood and MacConkey II agar plates**.** MacConkey agar plates were incubated for 48 hours at 37°C in an aerobic atmosphere. Chocolate and blood agar plates were incubated similarly supplemented with carbon dioxide. Anaerobic culturing was not performed. Cultures were assessed after two days. Bacterial identification was performed using standard biochemical laboratory procedures, according to the clinical and laboratory standards institute recommendations [7].

Antibiotic susceptibility testing was performed according to the protocols of the European Committee on Antimicrobial Susceptibility Testing [8] by microdilution on Mueller-Hinton and *Haemophilus* test medium with the Bauer-Kirby disk diffusion method (bioMérieux, Lyon. France). The isolated microorganisms were tested for susceptibility to a variety of antibiotics. Based on the minimum inhibitory concentration breakpoints each microorganism was classified as susceptible (S), intermediate susceptible (I) and resistant (R) to the agent in question. Isolates exhibiting resistance to 3 or more drug classes were defined as multidrug-resistant [9].

Categorical variables were expressed as number and percentages. Distribution of continuous variables was assessed using a histogram. Categorical variables were compared using the Chi-square test. A two-tailed p<0.05 was considered statistically significant. SPSS version 23 statistical software (SPSS, Inc., Chicago, IL, The USA) was used to perform the statistical analysis.

## RESULTS

In the current retrospective study, 191 samples from 191 children with acute bacterial conjunctivitis (89 males, 102 females) were analyzed. Seventy samples (36.6 %) had negative results for culture (Table 1). Flora was found in 8 samples (4.2%, 3 males, 5 females) - 3 in Group A and Group B and 2 in Group C. These results were not included in the analysis.

**Table 1:** Culture Results From Samples of Childhood Acute Bacterial Conjunctivitis

Result	Number of specimens	Percentage
Sterile culture	70	36.6
Flora	8	4.2
One organism	107	56
Two organisms	6	3.2

Microbial growth was detected in 113 bacteriological cultures (59.2%). More precisely, positive result for cultures was found in 34 neonates (Group A, 16 male and 18 female) and included the growth of 34 isolates. To be noted, all newborns were delivered by vaginal delivery. Positive results for culture were found in 57 patients (30 male and 27 female) of Group B with growth of 63 microorganisms. Finally, 22 samples from Group C children (8 male and 14 female) had positive results for culture which included the growth of equal number of isolates. Table 2 shows the distribution of the isolated pathogens in each group of patients. There was no statistical significant difference in the incidence of pathogens between the 3 age groups (P = 0.084). In neonates, over the half of isolates were coagulase negative *Staphylococcus* spp. The second more frequent pathogen was *S. aureus* followed by *S viridans*. In Group B the most frequent pathogens were several *Haemophilus* spp. followed by *Staphylococcus* spp. and in Group C coagulase negative *Staphylococcus* spp. and several *Haemophilus* spp. prevailed.

**Figure 1:**
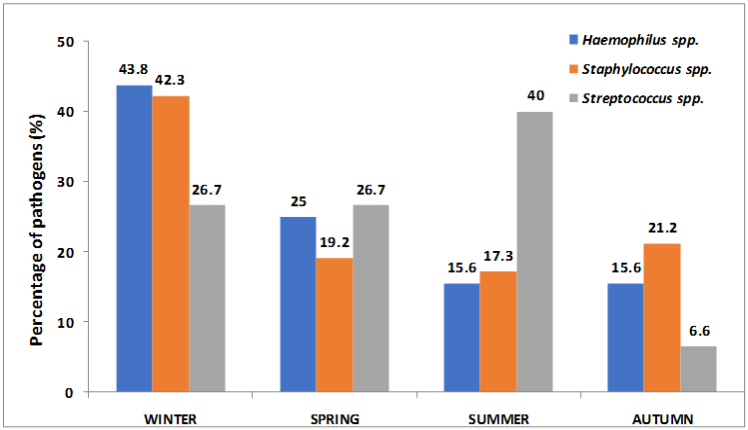
Seasonal Distribution of the 3 Main Pathogens Isolated From Specimens of Childhood Acute Bacterial Conjunctivitis

Seasonal distribution of the 3 main pathogens isolated was also recorded. Regarding *Haemophilus* spp. 43.8% of them were isolated during winter, while 25% in spring, 15.6% in summer and 15.6% in autumn. Also, 42.3% of *Staphylococcus* spp. were isolated during winter, 19.2% in spring, 17.3% in summer and 21.2% in autumn. Concerning *Streptococcus* spp. 26.7% of samples were identified in winter, 26.7% in spring, 40% in summer and 6.6% in autumn (Fig. 1). 

**Table 2:** Types and Number of Isolates Detected in Each Group with Childhood Acute Bacterial Conjunctivitis

Isolated microorganism	Group A	Group B	Group C
*Staphylococcus* spp.			
**Coagulase negative**	19 (55.9)	10 (15.9)	7 (31.9)
***S. aureus***	5 (14.7)	9 (14.3)	3 (13.6)
*Streptococcus* spp*.*			
***S. viridans***	4 (11.9)	1 (1.6)	1 (4.5)
***S. pneumoniae***	-	9 (14.3)	3 (13.6)
***S. pyogenes***	-	1 (1.6)	-
*Haemophilus* spp*.*			
***H. influenzae***	1 (2.9)	7 (11.1)	1 (4.5)
**Other **	2 (5.9)	17 (26.9)	6 (27.4)
***Corynebacterium Diphtheriae***	1 (2.9)	-	-
***Enterobacter cloacea***	1 (2.9)	1 (1.6)	-
***Moraxella spp.***	1 (2.9)	3 (4.7)	-
***Escherichia coli***	-	2 (3.1)	-
***Bacillus spp.***	-	1 (1.6)	-
***Pseudomonas aeruginosa***	-	1 (1.6)	1 (4.5)
***Serratia marcescens***	-	1 (1.6)	-

Group A: included neonates under 28 days of life; Group B: children from 1 month to 2 years; Group C: children from 2 years to 14 years; n: number; %: percentage

Antibiotics sensitivity was evaluated from some of the isolates. Table 3 shows the sensitivity from all the evaluated microorganisms as well as the sensitivity from some isolates of *S. aureus, Haemophilus spp.* and *Streptococcus spp*.

Sensitivity evaluation from all the examined microorganisms revealed highest resistance in ampicillin, ceftazidime, ceftriaxone and sulfamethoxazole. More precisely, of 49 evaluated microorganisms 44.9% were resistant to ampicillin. Furthermore, 20.4% of the 54 evaluated microorganisms were resistant to sulfamethoxazole. The resistance of all isolates to the most commonly empirically prescribed antibiotics in ophthalmology, i.e. tobramycin and ciprofloxacin were 14.3% and 2.3% respectively. However, only a small number of microorganisms were tested for resistance to tobramycin. *Staphylococcus aureus* exhibited the highest resistance to the following antibiotics; ampicillin (84.6%) and ceftazidime (40%). One of 3 *S. aureus* isolates evaluated were resistant to tobramycin and 9% of the 11 *S. aureus* isolates were resistant to ciprofloxacin. Highest resistance rates of *Haemophilus spp. *against antibiotics corresponded to ampicillin (28%) and sulfamethoxazole (24%). None of the isolated *Haemophilus spp. *exhibited resistance to ciprofloxacin. *Streptococci spp. *demonstrated the higher resistance against clarithromycin (15.4%) and sulfamethoxazole (11%). We did not find any vancomycin resistant isolate. Finally, we isolated 8 multidrug-resistant bacteria. One multidrug-resistant microorganism was isolated in Group A, 6 in Group B and 1 in Group C.

**Table 3:** Sensitivity of the Isolated Microorganisms in Childhood Acute Bacterial Conjunctivitis. The Numbers in the Parentheses Demonstrate Evaluated Number (n) of Isolates. (R: Resistant, S: Sensitive, I: Intermediate Resistant)

	MICROORGANISM			
Antibiotics	**All isolated microorganisms**	***Staphylococcus*** ***aureus***	***Haemophilus*** *** spp.***	***Streptococcus*** *** spp.***
	**(n) R%/S%/ I%**	**(n) R%/S%/ I%**	**(n) R%/S%/I%**	**(n) R%/S%/ I%**
Ampicillin	(49) 44.9%/ 55.1%/ 0%	(13) 84.6%/ 15.4%/ 0%	(25) 28%/ 72%/ 0%	(5) 0%/ 100%/ 0%
Cefuroxime	(60) 11.7%/ 88.3%/ 0%	(15) 13.3%/ 86.7%/ 0%	(31) 12.9%/ 87.1%/ 0%	(8) 0%/ 100%/ 0%
Ceftazidime	(24) 20.8%/ 70.8%/ 8.4%	(10)40%/ 40%/ 20%	(8) 12.5%/ 87.5%/ 0%	-
Ceftriaxone	(13) 23.1%/ 76.9%/ 0%	(4) 50%/ 50%/ 0%	(7) 14.3%/ 85.7%/ 0%	-
Sulfamethoxazole	(54) 20.4%/ 77.8%/ 1.8%	(13) 7.7%/ 92.3%/ 0%	(25) 24%/ 72%/ 4%	(9) 11%/ 89%/ 0%
Gentamicin	(14) 0%/ 100%/ 0%	(8) 0%/ 100%/ 0%	-	-
Amikacin	(16) 0%/ 93.8%/ 6.2%	(10) 0%/ 90%/ 10%	-	-
Netilmicin	(5) 0%/ 100%/ 0%	(5) 0%/ 100%/ 0%	-	-
Tobramycin	(7) 14.3%/ 85.7%/ 0%	(3) 33.3%/ 66.7%/ 0%	-	-
Clarithromycin	(58) 12.1%/ 87.9%/ 0%	(15) 6.7%/ 93.3%/ 0%	(29) 13.8%/ 86.2%/ 0%	(13) 15.4%/ 84.6%/ 0%
Vancomycin	(14) 0%/ 100%/ 0%	(6) 0%/ 100%/ 0%	-	(8) 0%/ 100%/ 0%
Ciprofloxacin	(44) 2.3%/ 97.7%/ 0%	(11) 9%/ 91%/ 0%	(20) 0%/ 100%/ 0%	(6) 0%/ 100%/ 0%

## DISCUSSION

According to our results, bacteriologic patterns of acute bacterial conjunctivitis in children remained similar to most previous studies with the *Staphylococcus* spp*.*, *Haemophilus* spp*.* and *Streptococcus* spp. being the main isolated pathogens [2, 3, 6, 10-14]. Their frequencies, however, vary significantly between studies. Carreras et al. reported that the most frequently isolated bacteria were *Staphylococci spp*. (56.6%), *Streptococci spp*. (21.4%) and *Haemophilus* *spp*. (12.1%). While, Patel et al. found that *H. influenzae* accounted for 73%, *S. pneumoniae* 14.4% and *S. aureus* 2% [3, 6]. The results of this study showed that in newborns, causative bacteria are inoculated from the genital tract and in older children vaccination against *Haemophilus spp.* and *Streptococcus spp.* does not affect the microbiological pattern of acute conjunctivitis. *Haemophilus* spp. were more frequently isolated in winter and *Streptococcus* spp. in summer. Contrary to expectations, antibiotic resistance rates in Western Greece were comparatively low. Our findings were broadly categorized in three age groups to investigate differences in causative pathogens between them. Newborns (<28 days of life) in our study, who were all delivered by the vaginal delivery method, were mainly infected by coagulase negative *Staphylococcus*, *Staphylococcus aureus *and *Streptococcus spp*. However, *Haemophilus spp*. were less frequent isolates in neonatal acute bacterial conjunctivitis. Our microbiologic results are typical for newborns, who are principally infected via oculogenital spread from the infected mothers, and in accordance with previous studies on the field, confirming the reported main pathogens, which are the predominant organisms colonizing the birth canal and environment [14, 15]. To examine the impact of vaccination on the microbiologic patterns of acute bacterial conjunctivitis older children were divided into two different groups. Regarding the most prevalent pathogens of acute bacterial conjunctivitis, the vaccination program in Greece includes vaccination with the *Haemophilus influenzae* type b (Hib) conjugate vaccine and 13-valent *Pneumococcal* conjugate vaccine (PCV13) until the second year of children’s life. According to our results, vaccination does not seem to affect the incidence of pathogens inflicting acute bacterial conjunctivitis since there were no statistically significant differences in the types of isolates between the age groups. This may be attributed to possible infection by other serotypes or nontypeable bacteria, known as "the replacement phenomenon" [16, 17].

Antibiotic resistance patterns observed in Western Greece in the current study exhibited a considerable variation compared to previous relevant studies [2, 3, 6, 17-19]. However, they seemed to be expectable due to differences in geographic areas, different periods of time or divergences in intensity of usage of several antibiotics [5, 20]. A comparative study also from Greece, and more precisely in the island of Crete in South Greece, found a decrease in resistance rates of *S. aureus* to several antibiotics from 2000 to 2009 [21]. More precisely, the resistance of *S. aureus* to tobramycin decreased from 29.4% to 14.5%, the resistance to chloramphenicol decreased from 25.5% to 18.8% and the resistance to ciprofloxacin decreased from 3.9% to 2.9%. Another study from Spain reported that in 2008 the resistance rates of the bacteria causing conjunctivitis against several antibiotics like tobramycin or gentamicin, increased significantly compared to 1982, while the resistance rates to chloramphenicol diminished significantly [6]. We found higher resistance rates against ampicillin, amoxicillin/clavulanate and sulfamethoxazole. Interestingly, low resistance rates were found against tobramycin and ciprofloxacin, two antibiotics that are widely used topically as empiric treatment in ophthalmology. Nevertheless, studies of bacterial conjunctivitis isolates conducted from the late 1990s through the mid-2000s have shown increased resistance to tobramycin. The first annual survey of Ocular Tracking Resistance in the United States Today (TRUST), describing data collected from October 2005 to June 2006 showed 65.3% resistance among *S pneumoniae* isolates to tobramycin and 63.6% among MRSA [22]. Furthermore, our results demonstrated rather low multidrug-resistant rates compared to other studies [2, 10, 17, 23, 24]. Regarding seasonal distribution, previous reports demonstrated that *Haemophilus* spp. are more frequently isolated in winter and *Streptococcus* spp. in summer, even corresponding percentages vary significantly [2, 25]. Block et al. [2] reported that 41.5% of total *H. influenzae* were isolated in winter, 33.8% of *S. pneumoniae* in summer, while Gigliotti et al. [25] reported that 40.5% of *H. influenzae* isolates were found in January and 52.3% from February to March. Our study demonstrated that most *Haemophilus spp*. (43.8%) and *Staphylococcus* *spp.* (42.3%) were isolated during winter while *Streptococcus* *spp*. were more prominent during summer (40%).

The long duration of our study (5 years), the large number of antibiotics studied and its initiative nature in our region give strength to our study. However, we had some limitations. Firstly, it was a retrospective study conducted in a single pediatric hospital, and we are not sure whether our results can be generalized to larger population groups. Furthermore, the sample size was small and we did not have sensitivity results from all the isolated microorganisms. A small number of isolates were tested against Tobramycin, whereas the newer quinolones were not included in the sensitivity evaluation. Finally, cotton-tipped swabs that were used in the current study contain fatty acids, which may inhibit bacterial growth. Specimen collection under topical anesthetic eye drops may minimize contamination and result in inhibitory effects on organisms recovered. However, we used this sample collection method to minimize any possible discomfort to studied pediatrics. 

We suggest to perform more investigations regarding antibiotic susceptibility patterns of pathogens implicated in acute bacterial conjunctivitis in children in our region with the collaboration of more hospitals and assessing more antibiotics in the antibiogram, especially of newer quinolones, to prepare more precise local guidelines for empirical therapy.

## CONCLUSIONS

The predominant pathogens of childhood acute bacterial conjunctivitis in Western Greece were *Staphylococcus spp., Haemophilus spp.* and *Streptococcus spp.* We did not find any statistically significantly differences in the isolated pathogens between the 3 age groups. Antibiotic resistance rates were relatively low. Low resistance rates against older antibiotics like tobramycin or ciprofloxacin highlight their significant role in the empirical treatment of childhood acute bacterial conjunctivitis. Continuous surveillance, focused in each geographic area, is encouraged to guide empirical treatment.

## DISCLOSURE

Ethical issues have been completely observed by the authors. All named authors meet the International Committee of Medical Journal Editors (ICMJE) criteria for authorship of this manuscript, take responsibility for the integrity of the work as a whole, and have given final approval for the version to be published. No conflict of interest has been presented.

## Funding/Support:

None.
